# Incidence of mycobacteria in pulmonary granulomatous lesions

**DOI:** 10.1016/j.clinsp.2024.100564

**Published:** 2024-12-28

**Authors:** Sibele Inácio Meireles, Mariana Vargas Cruz, Gustavo Palmer Irffi, Leonardo Abreu Testagrossa

**Affiliations:** Hospital Sírio Libanês, São Paulo, SP, Brazil

**Keywords:** Mycobacteria detection, Lung granulomatous lesions, FFPE specimens

## Abstract

Mycobacteria infections are caused by species of the *Mycobacterium tuberculosis* complex (MTB) and other species called Non-Tuberculosis Mycobacteria (NTM). Identification of mycobacteria species is very important to define treatment and it can be achieved by direct culture. However, the lack of clear protocols regarding the use of culture or molecular tests on specimens diagnosed with granulomatous lesions causes delays in the diagnosis of the etiological agents and, consequently, the definition of the right treatment. This work aimed to characterize the incidence of mycobacteria species in pulmonary granulomatous lesions and the contribution of Polymerase Chain Reaction (PCR) in Formalin-Fixed Paraffin-Embedded Tissue (FFPE), direct culture, and Ziehl-Neelsen histological stain to the diagnosis. The authors performed an observational, centralized, and retrospective study in a cohort of 336 cases with pulmonary granulomatous lesions. Mycobacteria were detected by ZNS in 54/323 (16.72 %) and by direct culture in 40/198 (20.20 %). MTB DNA was detected by PCR in 10/57 (17.54 %). Mycobacterial culture results revealed MTB in 26/40 (65.00 %), whereas NTM was detected in 13/40 (32.50 %). NTM was represented by *M. avium* (*n* = 4), *M. intracellulare* (*n* = 3), *M. kansasii* (*n* = 3), *M. colombiense* (*n* = 1), *M. paraffinicum* (*n* = 1), and *M. abscessus* subsp. *massiliense* (*n* = 1). In conclusion, this study demonstrated that mycobacteria are detected in 16.72 % to 20.20 % of pulmonary granulomatous lesions. Moreover, MTB and NTM were detected in these lesions. The use of different methods for mycobacteria detection, in addition to culture, is complementary and contributes to fastening and increasing the detection of mycobacteria in these lesions.

## Highlights

• Lung granulomas have been associated with mycobacteria and fungal infections.
• The largest cohort of pulmonary granulomatous lesions in Brazil was studied.
• FFPE specimens are a valuable source of material for molecular methods.
• Molecular methods contribute to the fast diagnosis of all mycobacteria species.

## Introduction

Lung lesions encompass a wide range of infectious and non-infectious conditions. Histological examination of lung lesions may reveal features, such as granulomatous inflammatory reactions or necrosis, offering indicators of mycobacteria infection in cases that had not previously raised suspicion. Granulomatous inflammatory reactions are a form of chronic response to various forms of injury, such as infectious, autoimmune, toxic, allergic, or neoplastic. In this type of lesion, there is the formation of aggregates of epithelioid macrophages, sometimes with multinucleated giant cells amidst a variable amount of inflammatory cells, such as lymphocytes. It may be accompanied by central necrosis, which is often related to infectious agents (most commonly *Mycobacterium tuberculosis* or fungi such as Histoplasma).^1^ In lung granulomatous lesions associated with mycobacteria, not only *Mycobacterium tuberculosis* (MTB) but also Non-Tuberculous Mycobacteria (NTM) can be the causative agent.^2^ Non-tuberculous mycobacteria are acid-fast bacilli that grow slowly in culture and are found in animals, water, and humid places.^3^^,^^4^ They account for a minority of mycobacterial infections; however, the number of patients with NTM has been increasing in recent decades.^3^^,^^5^ The involvement is mainly pulmonary, but there may also be infection of the lymphatic tissue, skin, subcutaneous tissue and bones. The symptoms are nonspecific and difficult to differentiate from tuberculosis in lung diseases.^2^ NTM pulmonary disease is usually associated with *Mycobacterium avium* complex (MAC), *Mycobacterium abscessus subsp abscessus*, and *Mycobacterium kansasii*.^3^

In clinical samples such as biopsies and body fluids, the differential diagnosis of MTB and NTM species is not possible through direct visualization, since both MTB and NTMs show positivity to the conventional smear acid-fast staining method (Ziehl-Neelsen). Mycobacteria species distinction is essential for choosing the right treatment and can be properly made by culture although it is time-consuming given the low growth rate. Therefore, the incidence of NTM is probably underestimated in many tuberculosis-endemic countries where there is limited access to ancillary tests needed to differentiate mycobacteria species.^6^, ^7^, ^8^, ^9^ Cultivating the mycobacteria in solid media is considered the gold standard for the diagnosis of mycobacteria.^10^ Detection of the right agent allows for the best treatment and prognostic assessment of the lesion. However, because of its low growth rate most of the time, it usually takes several weeks for the bacteria to be detected as visible colonies in solid culture media. Additionally, there are circumstances in which not enough material is collected for culture or it is not possible to detect any etiological agent in the specimen. Molecular diagnostic methods that offer rapid and specific results for mycobacteria detection are available commercially. One such method is DNA amplification through PCR, typically conducted using fresh samples, predominantly sputum. PCR assays seem to be adopted infrequently in routine diagnostics using Formalin-Fixed, Paraffin-Embedded (FFPE) tissue samples, possibly due to the increment in the cost for diagnosis and to limitations in the available assays when using FFPE specimens.

Although granuloma is a histological lesion pattern that has been classically described for a long time, the granuloma's etiological agent cannot always be precisely defined based solely on histological examinations, which remains a challenge in lung pathology. The authors hypothesize that distinct mycobacteria detection approaches demonstrate a high level of agreement and are complementary to one another. The objective of this work was to better elucidate the prevalence of mycobacterial infection according to different detection methodologies in pulmonary granulomatous lesions diagnosed at a large reference center hospital in Brazil. A correlation between the presence of necrotic granulomatous lesions and the associated mycobacteria species was also analyzed.

## Methods

**Fig. 1:**
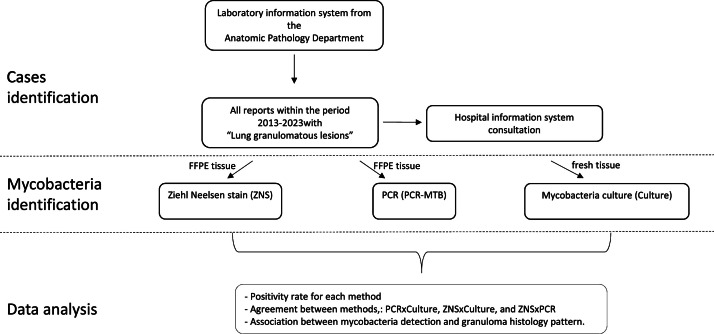
Study design.

In this observational, centralized, and retrospective study, the authors selected all cases with granulomatous lung disease based on the pathology report from the Anatomic Pathology Department at Hospital Sírio-Libanês in São Paulo, Brazil, between October of 2013 and June 2023. The study design is summarized in Fig. 1. The authors analyzed three hundred and thirty-six cases of granulomatous lung disease and their corresponding mycobacteria evaluation by PCR, Ziehl-Neelsen and/or culture. Review boards of the Institutional Research Ethics Committee of the Hospital Sírio-Libanês approved this study (approval number 6.304.558). Pathology reports available on the laboratory information system were reviewed to check the histological diagnosis and microscopic description of granulomatous lung disease, which included results of Ziehl-Neelsen Stain (ZNS) by light microscopy used for detection of Acid-Fast Bacilli (AFB). ZNS is performed manually using the ready-to-use HistoKit Ziehl-Neelsen Fite (Erviegas, Indaituba, SP, Brazil) or automatically using the Artisan Acid-Fast Bacillus (AFB) Stain Kit and the Artisan Link Pro Special Staining System (Agilent, Santa Clara, CA, USA). Following the staining, AFB is visible as red bacilli against a blue background indicating a sample positive for mycobacteria.

The authors used the patient´s electronic medical records to review the results of mycobacteria direct culture from fresh tissue, whenever available. Mycobacterial culture was conducted by inoculating samples in a Lowenstein-Jensen medium followed by up to 60 days of incubation. Samples are classified as positive or negative for mycobacteria in the report, and the identified species are recorded for positive cases.

The results of MTB DNA detection by Polymerase Chain Reaction (PCR-MTB) in FFPE tissue were also verified in the laboratory information system. The PCR-MTB is based on the MTB ELITe MGB® assay (ELITechGroup, Italy) that allows the detection of MTB (*M. africanum, M. bovis, M. bovis BCG, M. microti* and *M. canettii*). Reports indicated whether the analyzed specimen tested positive or negative for MTB DNA.

To identify potential bias in the comparison between different methods, the authors quantified the interval (in days) between specimen collection for culture and for histopathological analysis to determine whether the samples accurately reflected the current disease state. Similarly, FFPE specimens used for MTB DNA detection were verified to ensure they originated from the same histopathological evaluation.

The authors tested the hypothesis of high agreement between pairs of methods, considering the following pairs: (1) PCR-MTBxCulture, (2) ZNSxCulture (3) ZNSxPCR-MTB. The authors calculated the rate of agreement by dividing the number of concordant test results by the total number of tests. The authors also tested the null hypothesis of independence between the presence of necrosis and the presence of mycobacteria infection using either Pearson's Chi-Square or Fisher's exact tests, depending on the sample size. It was not considered missing data when Ziehl-Neelsen stain, PCR-MTB, and mycobacterial culture were not evaluated concomitantly in all cases, as suspicion of mycobacterial infection might arise from clinical characteristics, despite the detection of granulomatous lesions in histological analysis.

## Results

The authors identified in the laboratory information system three hundred and thirty-six cases with granulomatous lung disease diagnosed between 2013 and 2023. Demographic characteristics obtained from the cases are summarized in Table 1. The cases were classified according to the histological characteristics described in the diagnostic reports as follows: inflammatory or infectious (Not Otherwise Specified, NOS) (120 cases, 35.71 %) when no etiological finding was reported; fungal infection (118 cases, 35.12 %) when fungi were identified regardless of the presence of concomitant neoplasia; mycobacterial infection when ZNS, PCR-MTB and/or culture was positive for mycobacteria (72 cases, 21.43 %) regardless of the presence of concomitant neoplasia; and neoplastic disease without infection when granulomas were identified in the same specimen alongside concomitant neoplasia with no etiological agent described (26 cases, 7.74 %) (Table 1).

**Table 1:** Cases with granulomatous lesions in the lung.

Parameter	Description
**Age (year ± standard deviation)**	58.55 ± 16.72
**Sex, n (%)**	
Male	169 (50.30 %)
Female	167 (49.70 %)
**Histological classification, n (%)**	
Inflammatory or infectious (NOS)	120 (35.71 %)
Fungal infection	118 (35.12 %)^a^
Mycobacterium infection	72 (21.43 %)^b^
Neoplastic disease without infection	26 (7.74 %)
**Type of granuloma, n (%)**	
Necrotic	214 (63.69 %)
Non-necrotic	122 (36.31 %)
**Total, n (%)**	336 (100 %)

a *n* = 13, cases with concomitant neoplasia.

b *n* = 1, case with concomitant neoplasia.

Overall, 21.43 % of cases (72/336) with granulomatous lesions in the lung tested positive for mycobacteria (Table 1). The authors further analyzed the cohort with respect to each methodology used for mycobacteria evaluation. Mycobacteria analysis by direct culture in fresh tissue was performed in 198/336 cases (58.93 %) and the positivity rate was 20.20 % (40/198) (Table 2). The fresh material collected for culture was mainly lung tissue fragment (137/198, 69.2 %), followed by bronchoalveolar lavage (40/198, 20.2 %), pleura fragment (11/198, 5.6 %), sputum (5/198, 2.5 %), and lymph node (5/198, 2.5 %). Most samples (176/198, 88.89 %) were collected for culture within an interval of 2 days from the specimen collected for histopathological evaluation. Longer intervals were observed for the remaining 11.11 % (22/198) samples, as follows: 14 cases in the interval range of 3‒15 days; 6 cases in 16‒30 days; 1 case in 36 days, and 1 case in 76 days. MTB investigation by PCR in FFPE tissue was conducted in 57/336 cases (16.96 %) and the positivity rate using PCR-MTB was 17.54 % (10/57) (Table 2). FFPE specimens used for PCR-MTB originated from the same histopathological evaluation in nearly all cases (55/57, 96.49 %). There were only 2 cases were performed in lung tissue collected 2 and 20 days prior to the specimen diagnosed with lung granulomatous lesion. Finally, mycobacteria analysis by Ziehl-Neelsen Staining (ZNS) in FFPE tissue was evaluated on histopathological evaluation from most cases (323/336, 96.13 %) and demonstrated a positivity rate of 16.72 % (54/323) (Table 2).

**Table 2:** Positivity rate for mycobacteria.

Mycobacteria evaluation	Positivity rate n (%)
Culture	40/198 (20.20 %)
Ziehl-Neelsen stain (ZNS)	54/323 (16.72 %)
PCR (PCR-MTB)	10/57 (17.54 %)

Most culture-positive cases (26/40, 65 %) were identified as MTB whereas 13/40 (32.50 %) were NTM (Table 3). There was only one case (2.50 %) for which the direct culture result did not allow to determine the specific type of mycobacteria infection.

**Table 3:** Mycobacteria species detected by direct culture.

Group	Samples n (%)
MTB	26 (65.00 %)
*Mycobacterium tuberculosis*	

**NTM**	**13 (32.50 %)**
*Mycobacterium avium* (n=4)	
*Mycobacterium intracellulare* (n=3)	
*Mycobacterium kansasii* (n=3)	
*Mycobacterium colombiense* (n=1)	
*Mycobacterium abscessus* subsp. *massiliense* (n=1)	

**Mycobacterium not specified**	**1 (2.50 %)**

Total	40 (100 %)

**Fig. 2:**
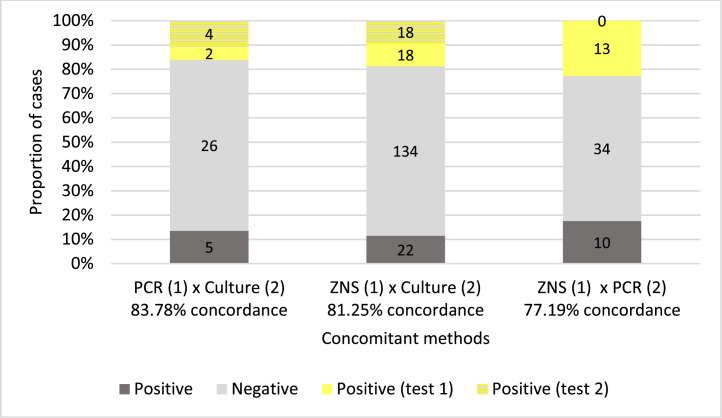
Summary of results in concomitant methods for mycobacteria detection. The results were compared between PCR-MTB (test 1) and Culture (test 2); ZNS (test 1) and Culture (test 2); ZNS (test 1) and PCR-MTB (test 2). Concordant results are presented in gray bars. Discordant results are indicated in yellow bars. ZNS, Ziehl-Neelsen Stain.

Results from the concordance analysis between pairs of mycobacterial detection methods are presented in Fig. 2. The concordance rates were 83.78 % for PCR-MTB versus culture, 81.25 % for ZNS versus culture, and 77.19 % for ZNS versus PCR-MTB, respectively. In the PCR-MTB versus culture comparison, NTM-positive cases in culture were excluded, as the PCR targeted only MTB. Discordant results were observed in all comparisons. For instance, in the PCR-MTB versus culture comparison, there were six discordances: two cases were positive by PCR-MTB only, while four cases were positive by direct culture only. On the other hand, in the ZNS versus PCR-MTB comparison, there were 13 discordant cases, all of which were positive by ZNS only. This discrepancy can be partly explained by the fact that ZNS detects all mycobacterial species (both MTB and NTM), while PCR-MTB detects only MTB.

**Fig. 3:**
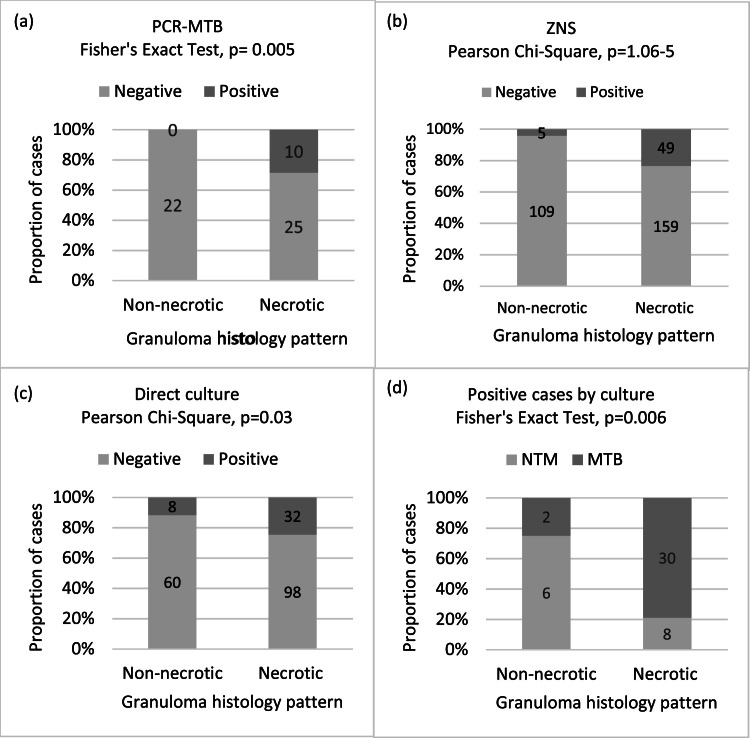
Association between mycobacteria detection methods and granuloma histology pattern. Necrotic granulomas are associated with mycobacteria positive results obtained by PCR-MTB (a), ZNS (b), and direct culture (c). Necrotic granulomas are significantly associated with the occurrence of MTB in culture (d). ZNS, Ziehl-Neelsen Stain.

Further analysis of the histology of granulomatous lesions, revealed that 63.69 % (214/336) presented with a necrotic pattern (Table 1) which was significantly associated with the detection of mycobacteria in PCR-MTB (Fisher's Exact Test, *p* = 0.005), ZNS (Pearson Chi-Square, *p* = 1.06^–5^), and direct culture (Pearson Chi-Square, *p* = 0.03) (Fig. 3a‒c). Interestingly, a significantly higher frequency of necrosis was observed in MTB positive cases (30/38) in comparison to NTM (8/38) (Fisher's Exact Test, *p* = 0.006) (Fig. 3d).

## Discussion

Granulomas are lesions that have been classically described histologically, however, their precise and detailed features associated with the presence of mycobacteria infection are not well defined. Tuberculosis is the most frequent cause of granulomatous lesions worldwide, however, other infectious causes, such as fungi and non-tuberculosis mycobacteria, also play a role. Therefore, the aim of this work is to determine the microbiological prevalence and features of mycobacteria infection in pulmonary granulomatous lesions diagnosed at a private hospital in São Paulo, Brazil. This study identified 336 granulomatous lung lesions diagnosed between 2013 and 2023 and that these lesions were associated with mycobacteria infection in 21.43 % of the cases (72/336) (Table 1). Similarly to this observation, a retrospective review of 500 lung biopsies and resections from institutions in 7 countries demonstrated that mycobacterial infection was diagnosed in 56 of 300 cases (19 %) outside the US versus 16 out of 200 (8 %) in the US.^11^ In the studied cohort, the mycobacteria detection rate based on the detection method used was 20.20 % (40/198), 16.72 % (54/323), and 17.54 % (10/57) for direct culture, ZNS in FFPE specimens, and PCR-MTB in FFPE specimens, respectively (Table 2).

The authors also observed that, among mycobacteria culture-positive cases, most of the granulomas (65 %) were associated with the presence of *Mycobacterium tuberculosis c*omplex (MTB) (Table 3). Non-Tuberculosis causing Mycobacteria (NTM) were present in 32.50 % of the mycobacterial associated granulomatous lesions with the detected species including *M. avium, M. intracellulare, M. kansasii, M. colombiense, M. paraffinicum*, and *M. abscessus* subsp. *massiliense*. The present finding is in accordance with a recent review article from Dos Anjos et al., describing that the main NTM species associated with lung infections are *M. avium* complex (which includes the subspecies *M. avium, M. intracellulare,* and *M. chimaera*) and *M. kansasii*.^12^ There are few reports of *M. colombiense* causing lung disease, as observed in the studied cohort, which included one lethal case.^13^^,^^14^ Nonetheless, based on molecular approaches, *Mycobacterium colombiense* has been assigned to the *M. avium* complex and may not always be described as a distinct entity in studies, but rather as part of the *M. avium* complex. The other NTM species observed in the present cohort (*M. paraffinicum*, and *M. abscessus* subsp. *Massiliense)* have also been described previously in pulmonary lung lesions and are considered of pathogenic potential. A case of symptomatic pulmonary infection in humans caused by *Mycobacterium paraffinicum* was first described in 2014.^15^ The detection of *M. abscessus* subsp. *Massiliense* in lung lesions within the cohort highlights the importance of accurately defining mycobacterial species, as this particular subspecies is notably resistant to antibiotic therapy.^3^^,^^16^

The histology of granulomatous lesions was reviewed and further characterized in the present study, particularly regarding the presence of necrosis. The authors observed that 63.70 % (214/336) of the lung granulomatous lesions were necrotizing which is morphologically suggestive of infectious granulomas (Table 1). Furthermore, the presence of necrosis was significantly associated with the detection of mycobacteria regardless of the method used for detection (Fig. 3). In accordance with the literature, necrotizing granulomas usually have an infectious etiology, most caused by mycobacteria or fungi.^1^ In this work, the authors observed that a significantly higher frequency of necrosis was observed in MTB-positive cases in comparison to NTM (Fig. 3d). Based on this observation, the authors can speculate that granulomas without necrosis but with ZNS positivity strongly suggest the presence of non-tuberculosis mycobacteria.

Another objective of this work was to better elucidate the prevalence of mycobacterial infection in pulmonary granulomatous lesions according to different detection methodologies. The authors observed a strong concordance among the methods used for mycobacteria detection, with percentages ranging from 83.78 % for PCR-MTB and 81.25 % for ZNS compared to culture, to 77.19 % when comparing ZNS and PCR-MTB (Fig. 2). However, the variable detection rates among these methods needs to be interpreted with caution, as they are influenced by differences in the types of specimens tested for each method. The authors did not find any previous studies comparing the concordance of these three methods in the context of lung granulomatous lesions on biopsies. However, similar to these findings, a comparison between PCR analysis on FFPE tissue with granulomatous lesions and culture results was reported previously for 41 cases with 83 % concordance (34/41).^17^ Additionally, a review study focusing on the use of molecular methods for the detection of microorganisms in granulomatous lesions emphasizes their utility in complementing the identification of the etiological agent, including several PCR methods for mycobacteria detection.^8^ The specimens used for the different mycobacteria detection methods analyzed in this study were not identical, which may have influenced the concordance between the methods. Moreover, positivity rates should be interpreted with caution, as the present study's cohort consists of patients who visit a private hospital in São Paulo, Brazil. This cohort likely represents a subpopulation with higher income levels than the general Brazilian population. However, the authors were not able to control this bias.

Ziehl-Neelsen stain have variable sensitivity reported in the literature, ranging from 8.3 % to 60 % in culture-positive cases.^1^ Similarly, amongst 40 direct culture-positive cases, Ziehl-Neelsen was positive in 55 % of the cases (22/40) (Fig. 2, ZNSxCulture).

In conclusion, this study demonstrates that mycobacteria is detected in 21.43 % of pulmonary granulomatous lesions from a large private reference hospital in São Paulo, Brazil. Mycobacteria infections in these lesions are caused by MTB (65 %) and NTM (32.5 %). Besides the usual NTM species that notoriously affect the lungs, the authors also observed an occurrence of *M. colombiense, M. paraffinicum*, and *M. abscessus* subsp. *massiliense* which has been rarely reported in the literature among cases of lung NTM infections. The use of methods for mycobacteria detection in FFPE tissue, such as PCR-MTB and ZNS, demonstrated a high concordance with culture (83.78 % and 81.25 %, respectively), and contributed to an increase in the detection rate of mycobacteria in granulomatous lesions. Anatomic pathology laboratories worldwide that conduct molecular pathology can incorporate a PCR panel for mycobacterial detection in FFPE tissue to enhance the identification of this infectious agent in suspected lesions. This tool can enhance cost-effectiveness and expedite mycobacteria detection in suspected cases. Moreover, expanded PCR panels for detecting NTM and MTB in FFPE specimens are recommended to improve diagnostic efficiency.

## Authors’ contributions

Sibele Inácio Meireles: Conception and design, data collection and curation, writing the original draft.

Mariana Vargas Cruz: Conception, statistical analysis, and original draft review.

Gustavo Palmer Irffi: Data curation, original draft review.

Leonardo Abreu Testagrossa: Critical revision of the manuscript.

## Funding

This study required no funding.

## Declaration of competing interest

The authors declare no conflicts of interest.

## References

[1] Rosen Y. (2022). Pathology of granulomatous pulmonary diseases. Arch Pathol Lab Med.

[2] Gopalaswamy R., Shanmugam S., Mondal R., Subbian S. (2020). Of tuberculosis and non-tuberculous mycobacterial infections ‒ a comparative analysis of epidemiology, diagnosis and treatment. J Biomed Sci.

[3] Winburn B., Sharman T. (2024). StatPearls [Internet].

[4] Rahama O., Thaker H.CME (2013). Infectious diseases Atypical mycobacteria : an important differential for the general physician. Clin Med (Lond).

[5] Forbes B.A., Hall G.S., Miller M.B., Novak S.M., Rowlinson M.C., Salfinger M. (2018). Practice guidelines for clinical microbiology laboratories: mycobacteria. Clin Microbiol Rev.

[6] Shah K.K., Pritt B.S., Alexander M.P. (2017). Histopathologic review of granulomatous inflammation. J Clin Tuberc Other Mycobact Dis.

[7] Tahseen S., Ambreen A., Ishtiaq S., Khanzada F.M., Safdar N., Sviland L. (2022). The value of histological examination in the diagnosis of tuberculous lymphadenitis in the era of rapid molecular diagnosis. Sci Rep.

[8] Guarner J. (2012). Detection of microorganisms in granulomas that have been formalin-fixed: review of the literature regarding use of molecular methods. Scientifica (Cairo).

[9] Nopvichai C., Sanpavat A., Sawatdee R., Assanasen T., Wacharapluesadee S., Thorner P.S. (2009). PCR detection of Mycobacterium tuberculosis in necrotising non-granulomatous lymphadenitis using formalin-fixed paraffin-embedded tissue: a study in Thai patients. J Clin Pathol.

[10] Gholoobi A., Masoudi-Kazemabad A., Meshkat M., Meshkat Z. (2014). Comparison of culture and PCR methods for diagnosis of Mycobacterium tuberculosis in different clinical specimens. Jundishapur J Microbiol.

[11] Mukhopadhyay S., Farver C.F., Vaszar L.T., Dempsey O.J., Popper H.H., Mani H. (2012). Causes of pulmonary granulomas: a retrospective study of 500 cases from seven countries. J Clin Pathol.

[12] Dos Anjos L.R.B., Parreira P.L., Torres P.P.T.S., Kipnis A., Junqueira-Kipnis A.P., Rabahi M.F (2020). Non-tuberculous mycobacterial lung disease: a brief review focusing on radiological findings. Rev Soc Bras Med Trop.

[13] Barretto A.R., Felício J.S., Sales L.H.M., Yamada E.S., Lopes M.L., da Costa A.R.F. (2016). A fatal case of pulmonary infection by Mycobacterium colombiense in Para State, Amazon Region, Brazil. Diagn Microbiol Infect Dis.

[14] Tang M., Zeng W., Qiu Y., Fang G., Pan M., Li W. (2023). Clinical features of rare disseminated Mycobacterium colombiense infection in nine patients who are HIV-negative in Guangxi, China. Int J Infect Dis.

[15] Chan A.W., Kabbani S., Staton G., Kraft C.S. (2014). Mycobacterium paraffinicum causing symptomatic pulmonary infection. J Clin Microbiol.

[16] Griffith D.E. (2014). Mycobacterium abscessus subsp abscessus lung disease: “trouble ahead, trouble behind…”. F1000Prime Rep.

[17] Schulz S., Cabras A.D., Kremer M., Weirich G., Miethke T., Bösmüller H.C. (2005). Species identification of mycobacteria in paraffin-embedded tissues: frequent detection of nontuberculous mycobacteria. Mod Pathol.

